# Supplementary material for: Smart Anticorrosion Coatings Based on Poly(phenylene methylene): An Assessment of the Intrinsic Self-Healing Behavior of the Copolymer
Source: Polymers (Basel). 2022 Aug 24;14(17):3457. doi: 10.3390/polym14173457 (PMC9460725; doi:10.3390/polym14173457)

Supplementary Materials

S.M. 1. Synthesis of 4-octyloxybenzyl chloride

4-Octyloxybenzyl chloride was synthesized as reported previously (4): 4-hydroxybenzaldehyde (0.12 mol, 14.8 g) was dissolved together with potassium hydroxide (0.13 mol, 7.3 g) in acetonitrile (240 mL) in a three-necked flask. The reaction was stirred at room temperature and heated to reflux at 90 °C. 1-Bromooctane (0.14 mol, 25 mL) was then added during 1 h and the reaction mixture was vigorously stirred overnight. Thereafter, the mixture was cooled to room temperature and quenched with 250 mL of water, transferred to a separatory funnel and extracted with hexane (200 mL). The organic layer was washed twice with 30 mL of NaOH solution (10% in mass) and finally with water (three times 30 mL). The organic phase was dried over anhydrous sodium sulfate and concentrated in a rotary vapor at 300 mbar (40 ◦C) giving 4-octyloxy benzaldehyde as a pale-yellow oil (25.1 g, 0.11 mol, yield: 70%). 1H NMR (CDCl3): δ = 0.88 (t, 3H, CH3), 1.19–1.55 (m, 10 H, 5 CH2), 1.74–1.84 (m, 2H, CH2), 4.01 (t, 2H, CH2O), 6.65–6.98 (d, 2H, Ar), 7.79–7.82 (d, 2H, Ar), 9.85 (s, 1H, COH) ppm. Afterwards, 4-octyloxy benzaldehyde (25.1 g, 0.107 mol) was dissolved in tetrahydrofuran (THF) (20 mL) and added dropwise to a stirred suspension of NaBH4 (4.9 g, 0.11 mol) in dry THF (70 mL) at 0 °C. The reaction mixture was then warmed up to room temperature and stirred overnight. The reaction mixture was quenched with 50 mL of water and 50 mL of ethyl acetate were added to extract reaction product rated, washed with water (three times, 30mL) and dried over anhydrous magnesium sulfate [17]. The solvent was then removed by evaporation at 11 mbar and 40 ◦C, giving a white solid that was dissolved in 5 mL of THF and poured into 400 mL of water under stirring. The white precipitate was filtered (cellulose filter) and the cake was dried under vacuum (0.7 mbar) to give 4-octyloxybenzyl alcohol as a white powder (0.08 mol, 20.14 g, yield: 80%). 1H NMR (CDCl3): δ = 0.86–0.91 (t,

3H, CH3), 1.20–1.49 (m, 10 H, 5 CH2), 1.73–1.82 (m, 2H, CH2), 3.93–3.95 (t, 2H, CH2OAr), 4.61 (s, 2H, CH2OH) 6.87–6.90 (d, 2H, Ar), 7.26–7.29 (d, 2H, Ar) ppm. C15H24O2 (236.35 g mol−1): calcd. C 76.23, H 10.23, found C 76.18, H 10.12. Subsequently, 4-octcyloxybenzyl alcohol (0.03 mol, 16.71 g) was dissolved in dichloromethane (240 mL) and thionyl chloride (0.086 mol, 6.26 g) was added to the solution dropwise at 0 ◦C. The mixture was then stirred at the same temperature during 2 h and the reaction was subsequently quenched with water (100 mL). The organic layer was extracted, washed with water (60 mL) and saturated aqueous sodium hydrogen carbonate (140 mL) and dried over anhydrous sodium sulfate. The dichloromethane was evaporated at reduced pressure (100 mbar at 40 ◦C) obtaining 4-octyloxybenzyl chloride as a yellow pale oil (0.016 mol, 4.16 g, yield: 54%). 1H NMR (CDCl3): δ = 0.89–0.93 (t, 3H, CH3), 1.31–1.49 (m, 10 H, 5 CH2), 1.75–1.84 (m, 2H, CH2), 3.94–3.98 (t, 2H, CH2OAr), 4.57 (s, 2H, CH2Cl) 6.87–6.90 (d, 2H, Ar), 7.29–7.32 (d, 2H, Ar) ppm. C15H24OCl (254.80 g): calcd. C 70.71, H 9.10, found C 70.92, H 9.22.

S.M. 2. Synthesis of copolymers of phenylene methylene and 4-octyloxyphenylene methylene

Copolymerization was carried out by adding 10 g (1.50 mL, 0.039 mol) 4-octyloxybenzyl chloride under nitrogen atmosphere to 44.2 g (0.35 mol) destabilized benzyl chloride in a 100 mL three-neck flask containing 0.32 g (0.39 mol) of bismuth (III) trifluoromethansulfonate. The mixture was then heated up to 60 °C under a constant nitrogen flow of 0.4–0.5 mL min^{-1} to avoid the produced HCl from reaction environment. Because of a dramatic increase of the viscosity during polymerization, after 3 h the temperature was risen up to 120 °C for 3 h and subsequently to 180 °C for 17 h in order to guarantee a constant stir. In process controls were performed by analyzing with ^{1}H-NMR samples aliquot from the reaction mixture over the reaction time in order to assess the completion of the reaction.

Afterwards, the product was cooled down to room temperature and dissolved in 10 mL THF. The THF solution was then poured into 400 mL methanol under vigorous stirring for 4 h. The obtained powder was filtered through a cellulose filter and dried under vacuum (10^{-2} mbar) for 12 h. As established by ^{1}H-NMR spectra of the product the copolymer containing 13.4% mol/mol of octyloxy repeat units was obtained (13.26 g) (^{1}H-NMR ($CDCl_3$): δ = 0.86–0.91 (t, 3H, CH_3), 1.20–1.49 (m, 10 H, 5 CH_2), 1.73–1.82 (m, 2H, CH_2), 3.93–3.95 (t, 2H, CH_2OAr), 4.61 (s, 2H, CH_2OH) 6.87–6.90 (d, 2H, Ar), 7.26–7.29 (d, 2H, Ar) ppm. $C_{15}H_{24}O_2$ (236.35 g mol^{-1}): calcd. C 76.23, H 10.23; found C 76.18, H 10.12.

S.M. 3. Preparation of coatings

All the coatings are prepared as reported in our recent works [9,10]. AA2024 sheets with 4 cm length were pretreated previously with abrasive papers of 300, 500, 800, 1200, and 4000 grit in order to obtain a clean and polished surface. Successively, the polishing residues were removed from the samples by immersion in ethanol in an ultrasonic bath (Banerlin, Berlin, Germany) for 5 min and the cleaned surfaces were dried under nitrogen atmosphere. Finally, the surfaces were coated by a primer layer of benzyltriethoxysilane by spin coating (3500 rpm, 30 s) and subsequently heated up to 100 °C for 1 min, to achieve the condensation of benzyltriethoxysilane to polysiloxanes, as described earlier [9]. Polymer coatings with thickness 30 μm and 50 μm were obtained applying 100 mg of PPM blend or PPM copolymer by using a hot press (120°C, 8 KPa) and steel spacers of the corresponding dimensions [9,10].

S.M. 4. Evolution of the Bode modulus plot in ACET experiments after artificial damage (black) and self-healing during the first EIS (yellow), and second, third and fourth cycle (green, violet, light. blue); the fitting data are represented by lines while the experimental values are represented by circles.

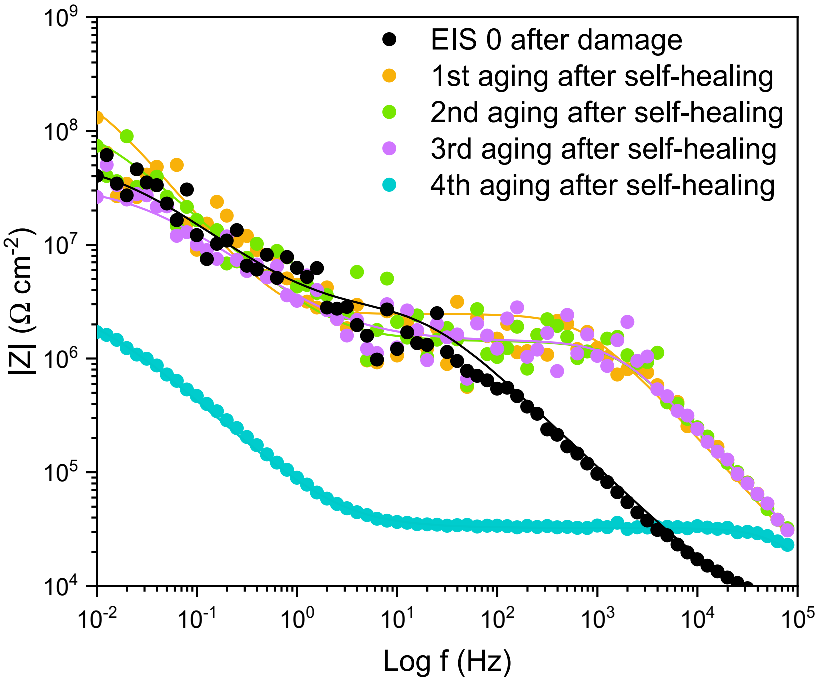

Supplement: Supplementary file 1 [file polymers-14-03457-s001.zip › polymers-1854201-supplementary.pdf]
